# mmContext: an open framework for multimodal contrastive learning of omics and text data

**DOI:** 10.1093/bioinformatics/btag338

**Published:** 2026-05-26

**Authors:** Jonatan Menger, Sonia Maria Krissmer, Clemens Kreutz, Harald Binder, Maren Hackenberg

**Affiliations:** Institute of Medical Biometry and Statistics (IMBI), Faculty of Medicine and Medical Center, University of Freiburg, Freiburg im Breisgau, 79104, Germany; Centre for Integrative Biological Signaling Studies (CIBSS), University of Freiburg, Freiburg im Breisgau, 79104, Germany; Institute of Medical Biometry and Statistics (IMBI), Faculty of Medicine and Medical Center, University of Freiburg, Freiburg im Breisgau, 79104, Germany; Centre for Integrative Biological Signaling Studies (CIBSS), University of Freiburg, Freiburg im Breisgau, 79104, Germany; Institute of Medical Biometry and Statistics (IMBI), Faculty of Medicine and Medical Center, University of Freiburg, Freiburg im Breisgau, 79104, Germany; Centre for Integrative Biological Signaling Studies (CIBSS), University of Freiburg, Freiburg im Breisgau, 79104, Germany; Institute of Medical Biometry and Statistics (IMBI), Faculty of Medicine and Medical Center, University of Freiburg, Freiburg im Breisgau, 79104, Germany; Centre for Integrative Biological Signaling Studies (CIBSS), University of Freiburg, Freiburg im Breisgau, 79104, Germany; Freiburg Center for Data Analysis, Modeling and AI, University of Freiburg, Freiburg im Breisgau, Germany; Institute of Medical Biometry and Statistics (IMBI), Faculty of Medicine and Medical Center, University of Freiburg, Freiburg im Breisgau, 79104, Germany; Centre for Integrative Biological Signaling Studies (CIBSS), University of Freiburg, Freiburg im Breisgau, 79104, Germany

## Abstract

**Summary:**

Multimodal approaches are increasingly leveraged for integrating omics data with textual biological knowledge. Yet there is still no accessible, standardized framework that enables systematic comparison of omics representations with different text encoders within a unified workflow. We present mmContext, a lightweight and extensible multimodal embedding framework built on top of the open-source Sentence Transformers library. The software allows researchers to train or apply models that jointly embed omics and text data using any numeric representation stored in an AnnData.obsm layer and any text encoder available in Hugging Face. mmContext supports integration of diverse biological text sources and provides pipelines for training, evaluation, and data preparation. We train and evaluate models for a RNA-Seq and text integration task, and demonstrate their utility through zero-shot classification of cell types and diseases across four independent datasets. By releasing all models, datasets, and tutorials openly, mmContext enables reproducible and accessible multimodal learning for omics–text integration.

**Availability and implementation:**

Pretrained checkpoints and full source code for our custom MMContextEncoder are available on Hugging Face huggingface.co/jo-mengr. The Python package github.com/mengerj/mmcontext provides the model implementation and training and evaluation scripts for custom training. The releases for the publication can be accessed via zenodo: adata_hf_datasets: doi.org/10.5281/zenodo.19185217 and mmContext: doi.org/10.5281/zenodo.19185493

## Introduction

The large body of annotated omics datasets, together with extensive biological knowledge, provides rich context for understanding cellular states. Recent work (Schaefer *et al.* 2025) on RNA-seq data has shown that such contextual information can be integrated by embedding textual descriptions of cells or samples using language models pre-trained on biomedical literature (Turc *et al.* 2019, Lee *et al.* 2020). Aligning these textual embeddings with gene-expression representations produces a joint latent space that combines complementary biological structure encoded in each modality, resulting in a more informative representation.

Existing implementations of such multimodal omics/text models remain difficult to customize and are not integrated into widely supported ecosystems such as Hugging Face. While foundation-model approaches are now used across omics domains, including single-cell and bulk transcriptomics, proteomics, and metabolomics, to learn generalizable biological representations (Jumper *et al.* 2021, Theodoris *et al.* 2023, Chen and Zou, 2024, Cui *et al.* 2024, Hao *et al.* 2024, Rosen *et al.* 2024, Hayes *et al.* 2025), there is currently no framework that enables direct comparison of different numeric and text-based representations of omics data within a single workflow.

To address these limitations, we introduce mmContext, a flexible multimodal embedding framework built on top of the Sentence Transformers library (Reimers and Gurevych 2019). The framework allows users to train or apply joint text–omics embedding models, using any numeric representation stored in an AnnData.obsm layer (Virshup *et al.* 2024) and any text encoder available on Hugging Face. Leveraging a mature ecosystem, mmContext inherits compatibility with diverse training regimes, multi-task learning, and downstream tools that rely on Sentence Transformer embeddings.mmContext enables systematic evaluation of different omics encoders, including Geneformer (Theodoris *et al.* 2023), scVI (Lopez *et al.* 2018), PCA, or gene-selection–based features. This allows users to tackle open questions in multimodal omics-text integration, such as whether pre-trained models provide more informative initial representations than simpler baselines such as a selection of genes. In addition to numeric encoders, mmContext supports text-based omics encoders that represent omics profiles as ranked feature lists (“cell sentences”), which can be directly embedded with a language model (Chen and Zou, 2024, Levine *et al.* 2024, Krissmer *et al.* 2025, Rizvi *et al.* 2025). To illustrate the capabilities of our framework, we focus on aligning RNA-seq and textual data for zero-shot cell-type and disease annotation, a challenging task where most existing methods rely on integrating test data into a reference dataset or applying a pre-trained classifier (Pasquini *et al.* 2021), and therefore cannot predict labels unseen during training. In mmContext, the language encoder provides a natural mechanism for zero-shot inference by embedding free-text label descriptions in the same latent space as expression profiles. This RNA-seq–based setting facilitates direct comparison with existing multimodal approaches such as CellWhisperer (Schaefer *et al.* 2025), while the framework remains broadly applicable to other omics–text integration tasks. We benchmark multiple model configurations, trained on large-scale pseudo-bulk and bulk RNA-seq data, systematically comparing pretrained omics embeddings with simpler numeric representations.

By releasing pretrained models and datasets on Hugging Face, mmContext provides a reproducible, open resource for multimodal exploration of single-cell and bulk transcriptomic data.

## Functionality

**Figure 1:**
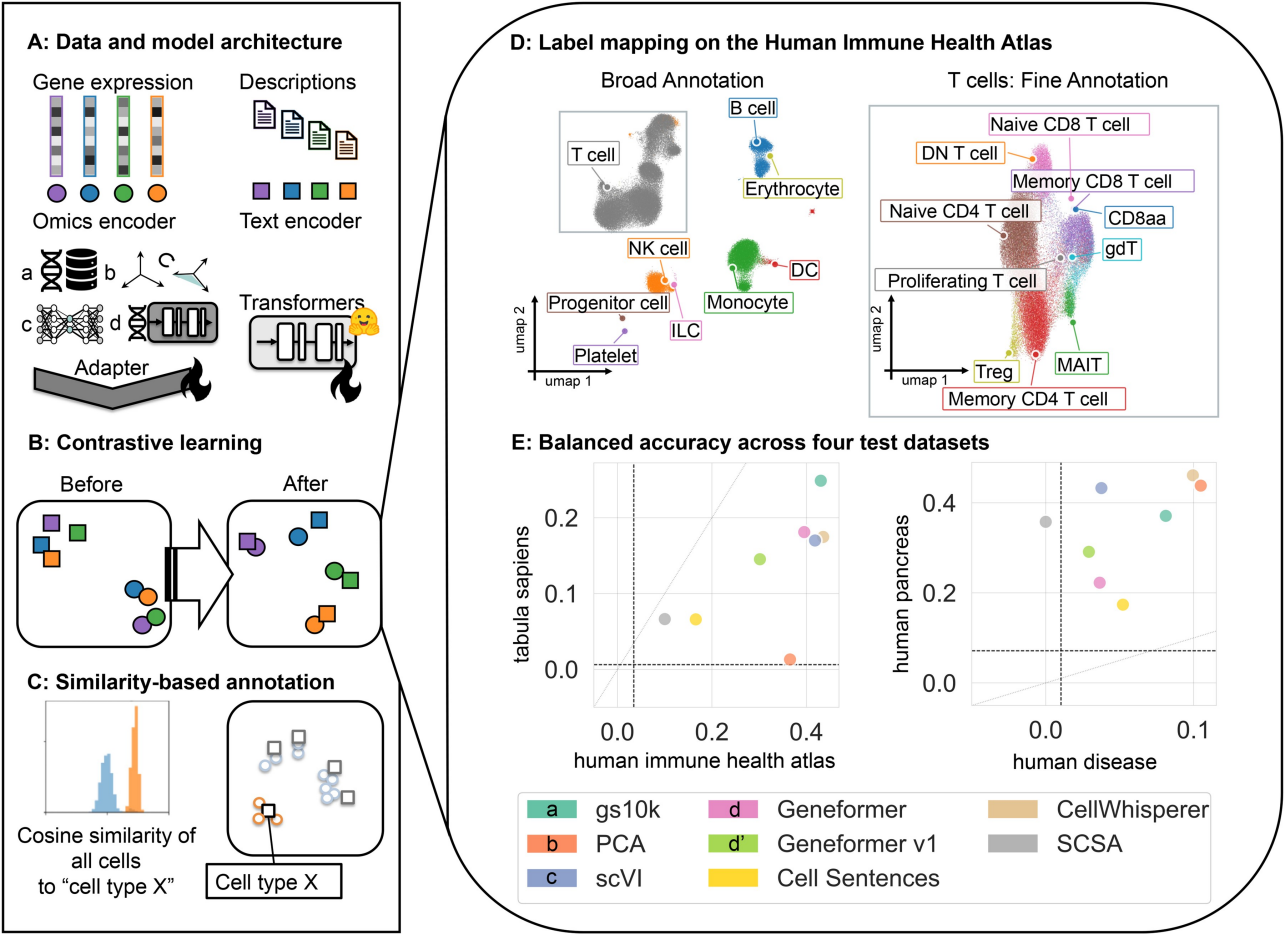
Overview of training and evaluation in mmContext. (A) Data and model architecture. Pairs of gene-expression profiles and corresponding text descriptions are passed through two modality-specific encoders. For the omics data, different encoders can be used to generate an initial numeric representation, which is optimized via an adapter layer. Currently implemented are: (a) a gene-selection embedder, (b) principal components of the selected genes, (c) embeddings from a pre-trained scVI model, and (d and d’) embeddings from a pre-trained Geneformer model with 104M parameters and 30M parameters. The text encoder can be any transformer model available on the Hugging Face Hub. (B) Contrastive learning. Omics and text modalities are aligned through a contrastive loss that increases similarity between matching pairs and decreases it for non-matching pairs. (C) Similarity-based annotation. After training, any text query (e.g., a cell-type label) can be embedded into the shared space to identify the most similar omics samples by cosine similarity. (D) Label mapping on the Human Immune Health Atlas. UMAP projection of the joint latent space shows well-aligned broad and fine-grained immune cell annotations on unseen test data. Created with the gs10k model, on a subset of 100k cells. (E) Balanced accuracy across four test datasets. Each point represents the balanced accuracy on two datasets achieved by a certain model; the dotted line indicates the random baseline ($1/n_{\text{classes}}$) accuracy per dataset. Besides the four initial omics encoders shown in A, a model using “cell sentences,” where count data is transformed into a list of gene names, is trained and evaluated with the mmContext framework. These mmContext models are compared to the CellWhisperer model by Schaefer *et al*. (2025) and a marker-based celltype annoation tool, SCSA (Cao *et al*. 2020).

The mmContext framework provides a modular interface for training and applying multimodal embedding models that align structured omics features with text descriptions in a shared latent space. Implemented as a custom model within the Sentence Transformers framework (Reimers and Gurevych 2019), it employs two modality-specific towers to encode omics and text data and optimizes similarity between true pairs while minimizing similarity between mismatched ones. Different dataset types and contrastive-learning objectives available in Sentence Transformers can be used without modification. An overview of the training and evaluation workflow is shown in Fig. 1.

Although this work demonstrates the approach using RNA-seq data, the same architecture can be directly extended to other omics modalities that can be represented as numeric feature vectors.

Below we outline the training workflow and resources available for working with the mmContext model: (A) Create initial omics representations and descriptions for each sample and store them in an AnnData object. (Virshup *et al.* 2024) Currently four omics encoders are implemented, which are detailed in the supplementary material, available as supplementary material at *Bioinformatics* online. The pairs are used to create a Hugging Face dataset, suitable for training with the Sentence Transformers Trainer. Tutorials and a complete pipeline for these steps are provided at github.com/mengerj/adata_hf_datasets. The workflow can be run locally or on an SSH server and supports out-of-memory chunking for large datasets. Users can also extend the pipeline by adding their own embedding method via a custom “InitialEmbedder.” Detailed instructions are available in the repository README. (B) Train the mmContext model with a contrastive learning objective, aligning pairs of omics representation and description. (C) Use the trained model to embed a new dataset and query it with free text. Any free-text query can be embedded in the joint space to compute similarity scores against the omics embeddings, enabling flexible analyses such as text-guided exploration or zero-shot classification. For classification, provide a set of candidate labels, embed each label string, compute pairwise similarities, and assign each sample the label with the highest score.

Prior to generating the initial embeddings, all datasets undergo quality control, normalization and log-transformation, following standard single-cell workflows (Luecken and Theis 2019), including filtering of low-quality cells and genes and batch-aware selection of highly variable genes. These preprocessing steps can be executed automatically before embedding generation and dataset conversion.

Because mmContext inherits the full Sentence Transformers API, users can easily integrate any text encoder available on the Hugging Face Hub (e.g., PubMedBERT, all-MiniLM, or Qwen-Embedding). Additional text-based datasets, such as NCBI abstracts (Krissmer *et al.* 2025), ontology definitions, or cell-type descriptions, can be incorporated to enrich the semantic structure of the joint embedding space and improve biological context alignment.

To demonstrate the framework’s capabilities, we trained mmContext with large-scale pseudo-bulk and bulk RNA-seq datasets that were also used in the CellWhisperer project, facilitating comparability. We showcase how different initial representations can be combined within the same workflow and evaluated on independent benchmarks for zero-shot cell-type and disease classification (Fig. 1D and E).

## Application example

We trained mmContext using the pseudo-bulk CellxGene dataset (approx. 20 million cells aggregated into 0.3 million pseudo-bulk profiles) and half the samples from the GEO bulk RNA-seq dataset (approx. 0.35 million samples) curated by Schaefer *et al.* (2025). Natural-language captions for each sample were generated from metadata, titles, and abstracts using a large language model as described in their work. For evaluation, we used four independent datasets not included in training: (i) the Human Immune Health Atlas with hierarchical cell-type annotations (Gong *et al.* 2024, Gustafson *et al.*  2024), (ii) Tabula Sapiens v1 containing over 150 human cell types (Consortium* *et al.* 2022), (iii) the Human Pancreas single-cell dataset with pronounced batch effects (Baron *et al.* 2016, Grün *et al.* 2016, Xin *et al.* 2016, Lawlor *et al.* 2017, Luecken and Theis, 2019), and (iv) a bulk RNA disease dataset with more than 100 disease labels (Schaefer *et al.* 2025).

All test data were unseen during training. For the experiments shown, we used PubMedBERT as the text encoder, producing 768-dimensional embeddings (NeuML 2023). Both omics and text encoders were mapped into a shared 2048-dimensional space via two separate two-layer networks. Training employed a contrastive loss (MultipleNegativesRankingLoss) (Henderson *et al.* 2017) on paired pseudo-bulk profiles and text captions using explicit and in-batch negatives. Further details on the training procedure, training time, and memory usage are provided in Supplementary Sections S4 and S5, available as supplementary material at *Bioinformatics* online.

In the experiments, we primarily assessed different omics encoders within the mmContext framework, allowing us to focus on the effect of the omics representation while keeping remaining components fixed. As a multimodal baseline we included CellWhisperer, which similarly aligns transcriptomic data with biological text, and additionally evaluated Single Cell Subtype Annotation (SCSA) (Cao *et al.* 2020) as a representative marker-based cell-type annotation approach.

The joint embedding space (Fig. 1D), obtained with the gs10k-based model, demonstrates good alignment between textual labels and cell-type embeddings, even for T-cell subtypes. Zero-shot classification was performed by comparing each test sample’s embedding to all label embeddings using cosine similarity, with predictions assigned by nearest-neighbor matching (Fig. 1C). We used balanced accuracy (Fig. 1E) as the primary evaluation metric. Despite the difficulty of multi-class datasets such as Tabula Sapiens, the best mmContext model (gs10k) achieved a performance of 40-fold above random baseline.

Across all benchmarks, models initialized with selected-gene embeddings (gs10k) on average performed better than those based on pretrained omics embeddings, demonstrating that biologically informed yet lightweight representations can rival large foundation-model encoders (see also Supplementary Section S6, available as supplementary material at *Bioinformatics* online). These results illustrate how mmContext enables reproducible comparison of diverse text-omics alignment strategies within a single modular framework.

As a complementary measure of ranking quality, the gs10k model achieved an AUC of 0.98 for broad immune lineages and 0.92 for fine grained annotations (see also Supplementary Tables 2–3, available as supplementary material at *Bioinformatics* online for all other evaluated models). To account for class imbalance in large cell-type annotation tasks, we additionally evaluated macro-F1, mean reciprocal rank, and top-k accuracy (Supplementary Tables 2–6, available as supplementary material at *Bioinformatics* online). The relative performance of the different omics encoders remained relative consistent across metrics for most models and datasets and confirms the trends observed in Fig. 1E (Supplementary Figures S1-S5, available as supplementary material at *Bioinformatics* online).

The Geneformer models and the cell-sentence approach are consistently outperformed by the remaining models across all evaluated tasks. While scVI demonstrates relatively strong performance across datasets, it falls short on the disease classification task. The PCA approach exhibits the weakest performance on the Tabula Sapiens dataset, yet achieves the highest performance on the disease classification task. The CellWhisperer model showed consistently strong performance across datasets. The marker-based tool SCSA was outperformed on the tabula sapiens and the human immune health atlas datasets. It showed competitive performance on the human pancreas dataset and could not be evaluated on the human disease dataset, as it can only predict cell types and not diseases.

All datasets and pretrained models used in this evaluation are publicly available on the Hugging Face Hub (https://huggingface.co/jo-mengr), allowing full reproducibility of the presented results and further experimentation within the mmContext framework.

## Conclusion

We introduced mmContext, a practical framework for aligning transcriptomic profiles with natural-language descriptions through contrastive learning built on the Sentence Transformers library. Systematic benchmarking shows that biologically informed gene-selection embeddings provide an effective numeric representation for multimodal alignment, outperforming current pretrained RNA-seq models.

Our findings highlight contrastive multimodal alignment as a viable foundation for zero-shot cell-type classifiers, while revealing remaining challenges in fine-grained subtype recognition. Future developments may include: (i) specialized cell-type models trained exclusively on single-cell data or domain-specific subsets such as immune cells; (ii) incorporation of additional omics modalities to construct richer joint embedding spaces for unpaired data; (iii) applying the model as a classifier or guiding signal for generative diffusion models of transcriptomes; and (iv) integration of mmContext into interactive, text-driven exploration tools for single-cell datasets.

By openly releasing both the framework and pretrained models, together with standardized datasets on the Hugging Face Hub, we provide a reproducible and extensible resource for the community and a foundation for future advances in language-aware multimodal omics analysis.

## Supplementary Material

btag338_Supplementary_Data

## Data Availability

Both the training data and the test data are publicly accessible through Hugging Face huggingface.co/jo-mengr, in a format that can be directly utilized for training with the SentenceTransformer based mmContext model. The training data was originally collected in Schaefer *et al.* (2025) and our preprocessed versions are stored on Zenodo, with links to each full dataset contained within the respective Hugging Face dataset.
