# Supplementary material for: mmContext: an open framework for multimodal contrastive learning of omics and text data
Source: Bioinformatics. 2026 May 26;42(6):btag338. doi: 10.1093/bioinformatics/btag338 (PMC13271244; doi:10.1093/bioinformatics/btag338)
Supplement: btag338_Supplementary_Data [file btag338_supplementary_data.pdf]

Supplementary material for mmContext: an open framework for multimodal contrastive learning of omics and text data

Jonatan Menger[1,2], Sonia Maria Krissmer[1,2], Clemens Kreutz[1,2], Harald Binder[1,2,3], Maren Hackenberg[1,2]

[1] Institute of Medical Biometry and Statistics (IMBI), Faculty of Medicine and Medical Center, University of Freiburg, Germany
[2] Centre for Integrative Biological Signaling Studies (CIBSS), University of Freiburg, Germany
[3] Freiburg Center for Data Analysis, Modeling and AI, University of Freiburg, Germany

* *Corresponding authors:* `jonatan.menger@proton.me` and `maren.hackenberg@embl.de`

Contents

S1 Preprocessing 2

S2 Implementation Details 2

S3 Gene Selection 3

S4 Training Details 4

S5 Time, computational and memory costs 5

S6 Results details 6

S7 SCSA Baseline 14

S8 Usage of LLMs 14

S9 References 14

S1 Preprocessing

The training data consists of a collection of publically available (pseudo-)bulk RNA sequencing profiles. This data was gathered and annotated with natural language descriptions by Schaefer et al. (2024). They gathered roughly 350 thousand pseudo-bulk samples from the cellxgene database (CZI Cell Science Program et al., 2025), by taking the mean expression of cells from the same biological context. The second dataset comprises bulk RNA-seq profiles from the NCBI GEO database (Barrett et al., 2005). All natural language descriptions were created with an LLM being prompted with metadata, available titles and abstracts (see Schaefer et al. (2024) for details). Cells with more than 3 MADs difference to the median percent of mitochondrial counts or more than 8% of mitochondrial genes are also removed. Genes present in less than 20 cells are removed and cells with less then 200 non-zero genes are also removed. Cells from low-frequency categories of interest (e.g., batch or cell type) with fewer than five occurrences in the dataset were excluded. To improve the models ability to correct strong batch effects, originating from different sequencing platforms, the instruments used were added to the caption of each cell. Raw counts are log-transformed and normalized. Batch aware highly variable gene selection is performed. Batches are considered to be different studies. Since a strong bimodal split was observed for the amount of aligned reads within the RNA-seq dataset gathered from the GEO database, and this effect dominated the variance of the dataset, we decided to split the dataset according to this bimodal distribution. Each split was then normalized separately. The origin of the split could not be resolved with the available metadata, but it is likely a technical artifact and not a biological one.

S2 Implementation Details

The Sentence Transformers framework (Reimers et al., 2019) trains dual-encoder models to learn similarity-based embeddings using paired data, maximizing cosine similarity for positive pairs and minimizing it for negatives. For textual input, a tokenizer maps text to token IDs, and a lookup table converts these IDs to embedding vectors.

To enable multimodal learning with numerical omics data, mmContext extends this architecture by introducing a modality-specific mechanism for registering continuous feature vectors (such as gene, protein, or metabolite profiles) as "tokens." Users provide a table that maps unique string identifiers to initial omics embeddings, from which the model constructs a frozen embedding matrix analogous to a text token embedding table. Each identifier can correspond to a cell, sample, or other omics entity, and the associated numeric representation may be defined at either the sample level (e.g., selected genes, PCA features, or embeddings from pretrained RNA-seq models) or the feature level (e.g., a protein-language-model embedding of the amino-acid sequence of a transcript, gene, or protein).

Because these omics embeddings are continuous and sample-specific rather than drawn from a fixed vocabulary, the model cannot rely on a persistent token dictionary as is done for text. Instead, the corresponding embeddings must be supplied whenever a new dataset is used. In practice, initial embeddings are stored in an AnnData object (Virshup et al., 2024) and loaded into the model at inference time through the mmContext API, ensuring that the embedding table is consistent with the method used during training.

At runtime, all inputs are handled as strings annotated with modality-specific prefixes. When a string begins with a registered omics prefix, the omics tokenizer retrieves the correct initial embedding from the frozen embedding matrix. Text strings are processed with the chosen transformer

tokenizer as usual. This design preserves full compatibility with the Sentence Transformers input pipeline while enabling fast lookup of numeric omics embeddings and seamless integration of mixed omics-text inputs within a shared latent space.

The currently implemented initial embedders are described in the following. (a) Gene selection (gs10k). Uses the expression matrix of 10,000 selected genes, including all known marker genes from CellMarker and PanglaoDB (details below). Genes absent from a dataset are set to zero. (b) Principal components. Obtained by applying PCA to the selected genes, reducing dimensionality to 50. The transformation is fitted jointly on subsets of the training data from CellxGene and GEO (roughly 80k samples from each dataset)(CZI Cell Science Program et al., 2025; Barrett et al., 2005), and the same loadings are reused for test data to ensure consistency between training and evaluation. (c) scVI embeddings. Derived from a pre-trained scVI model trained on the CellxGene corpus, producing a 50-dimensional embedding space (Ergen et al., 2025). In the following, this representation is referred to as scvi_fm (foundation model). (d) Geneformer embeddings. Obtained from the transformer-based Geneformer model (Theodoris et al., 2023), pre-trained on approximately 104 million cells and comprising 104 million parameters. The generated cell embeddings have 768 dimensions. Additionally, the Geneformer-v1 was used (d'), derived from the first version of the Geneformer model used in the CellWhisperer project, trained on approximately 30 million cells and producing 512-dimensional embeddings.

As an additional text-based input representation, cell sentences can be used, obtained by converting each expression profile into a ranked list of the most highly expressed genes which can be directly processed by a text encoder (Rizvi et al., 2025; Levine et al., 2024), jointly with the accompanying textual descriptions.

S3 Gene Selection

To define a compact but biologically relevant gene set, we combined reference gene annotations from GENCODE v49 (GRCh38.p14) with transcriptomic measurements from the Human Protein Atlas (HPA) consensus RNA dataset. The latter integrates RNA-seq data from HPA and GTEx across 50 consensus tissues, reporting normalized transcript counts (nTPM) for each gene. GENCODE annotations were parsed at the gene level to obtain Ensembl identifiers, HGNC symbols, and biotypes. To ensure compatibility with HPA data, Ensembl gene identifiers were normalized by removing version suffixes (e.g. ENSG00000141510.18 $\rightarrow$ ENSG00000141510). We restricted the gene universe to selected biotypes that are most relevant for transcriptomic analyses: protein coding, lincRNA, antisense, and processed transcript. Genes annotated as ribosomal RNAs (e.g. rRNA, Mt_rRNA), tRNAs, or pseudogenes were excluded. From the HPA consensus file (rna_tissue_consensus.tsv.zip), we generated a gene × tissue expression matrix using nTPM values. For genes with multiple entries per tissue, the maximum nTPM was retained, consistent with the consensus definition. Genes were required to be expressed above a detection threshold ($\geq$1 nTPM) in at least five tissues to minimize inclusion of spurious low-abundance transcripts. For each gene, three statistics were computed across the 50 tissues: 1. Mean expression (μ) 2. Variance of expression (σ^2) 3. Tissue specificity (Tau), defined as

$$\tau = \frac{\sum_{i=1}^{n}(1 - x_i / \max_j x_j)}{n-1} \quad (1)$$

where x_i denotes expression in tissue i. Tau ranges from 0 (ubiquitous expression) to 1 (restricted to a single tissue).

Each statistic was standardized (z-scored) across all genes, and a composite relevance score was calculated as

$$\text{score} = 0.5 \cdot z(\sigma^2) + 0.4 \cdot z(\tau) + 0.1 \cdot z(\mu). \tag{2}$$

This weighting prioritizes genes with high variability and tissue-specific patterns, while retaining moderately expressed ubiquitous genes.

Genes were ranked by the composite score and the top 10,000 were retained as the analysis panel. To ensure inclusion of well-established marker genes (e.g. immune and developmental markers), we additionally unioned curated marker sets from CellMarker and PanglaoDB with the ranked list. Duplicate entries were resolved by gene symbol, and the resulting panel contained both protein-coding genes and non-coding RNAs with established or putative biological roles.

This approach balances breadth and relevance: it avoids the inflation caused by tens of thousands of non-coding loci in GENCODE, yet preserves tissue-specific and regulatory genes (including lncRNAs) that may be critical for cell identity and disease.

S4 Training Details

After processing the training data, including the creation of the initial embeddings, datasets were stored on huggingface. These datasets contain both a cell token, which is simply the cell identifier, a cell sentences of 4096 genes, which can be truncated at training time and a path poiniting to the adata object, which can also be a share link to a cloud store. For all shown models, training is performed for 16 epochs with a batch size of 512, while keeping the text encoder frozen for the first epoch, thus only optimizing the adapter layers. Two types of negatives are used. The first are construction-time negatives, created during dataset building: For each anchor sample, negatives were drawn from other samples within the same experimental batch: one negative caption and one negative numerical representation, each taken from a different sample. Sampling negatives from both modalities strengthens cross-modal integration, and drawing them from the same experimental batch improves batch integration. The second type are training-time negatives, sampled on-the-fly from the same mini-batch during training. Together, these negatives are leveraged via the MultipleNegativesRankingLoss from the SentenceTransformers library (Henderson et al., 2017).

Negatives are therefore partly implicit, as the multiple negatives ranking loss treats all other anchor–positive pairs in the same batch as negative examples. This in-batch sampling assumes that most samples within a batch correspond to different labels, which holds given the large and diverse training dataset. Using in-batch negatives substantially increases the number of contrastive comparisons without requiring explicit negative sampling, improving training efficiency and stability. At the same time, the construction-time sampled negative samples support multi-modal and across-batch integration.

All shown models were trained with the PubMedBert-Embeddings model, which we found to work well for this task, while being computationally light. (NeuML, 2023) The training parameters, including the output dimension, were mainly chosen to match those used to train the CellWhisperer model, in order to facilitate comparison.

S5 Time, computational and memory costs

Table 1: **Computational requirements for different training runs.** This training time assumes initial embeddings were already constructed. The significant longer training time of the gs10k model can be explained due to the much larger input size. While the other models receive input vectors of dimensions 50 or 512, the gs10k model receives a sparse vector of 10 000 genes. This leads to a larger feedforward layer, with more trainable parameters. While the training time is longer at this step, there is no need to create initial embeddings beforehand, which is a significant advantage over the other methods, which require further compute during that first step. Values were recorded with the Weights & Biases tool (Biewald, 2020).

Text Encoder	Omics Encoder	GPU	RAM (GB)	VRAM (GB)	Epochs	Batch Size	Training Time
NeuML/pubmedbert-base-embeddings	gs10k	H100	41.9	97.9	16	512	31h 14m
NeuML/pubmedbert-base-embeddings	PCA	H100	44.3	43.8	16	512	11h 44m
NeuML/pubmedbert-base-embeddings	scVI	H100	48.5	43.9	16	512	12h 3m
NeuML/pubmedbert-base-embeddings	Geneformer	H100	45.1	46.1	16	512	11h 40m
NeuML/pubmedbert-base-embeddings	Geneformer v1	H100	43.2	45.3	16	512	11h 43m
NeuML/pubmedbert-base-embeddings	Cell Sentences	H100	53.0	39.1	16	512	21h 19m

S6 Results details

To provide a more nuanced view of the performance of different embedding strategies while taking into account the class imbalance in the datasets, we evaluated macro F1 scores, mean AUC, mean rank reprocity and top-k accuracy for $k = 1, 3, 5$ as supplementary metrics in addition to the balanced accuracy metric shown in Figure 1E. The results are reported in Tables 2-6.

On the human immune health atlas, both broad and fine-grained label annotations were used. On the broad annotation (AIFI_L1), the scVI and the PCA intial embedders outperform the other methods slightly, but the difference to the gs10k embedder is marginal. On the fine-gained annotations, the gs10k embedder performs best on 4 out of the 7 metrics. The geneformer embedder shows a strong top-1-accuracy, but the low balanced accuracy score suggest this to be caused by class imbalance.

The human disease dataset is particularly challenging for all investigated models, with rather low balanced accuracy and macro F1 values throughout. Yet, the AUC values of >0.8 for PCA and gs10k based models point towards the general feasibility of the mmContext approach for annotating disease information and the task would likely benefit from more explicit training data. This is consistent with the composition of the training data, which contains comparatively limited disease-specific information as most cells are obtained from healthy individuals.

The human pancreas dataset is challenging due to the strong batch effects in the data. CellWhisperer performs best on balanced accuracy, mean reciprocal rank, and all top-k accuracy metrics, making it the strongest overall model on this benchmark. Among the mmContext variants, the PCA- and scVI-based model perform best, followed by the gs10k embedding. Notably, the marker-based SCSA baseline is competitive on this dataset and even achieves the highest overall macro F1 score, although ranking-based metrics are not available for this method.

Lastly, on the tabular sapiens dataset, which includes over 150 cell-type labels, the gs10k based mmContext model shows the strongest performance in balanced accuracy, macro F1 score, and top-1 accuracy, while the scVI-based model perform marginally better on ranking-oriented metrics. The PCA-based model performs poorly on this dataset, indicating that the reduced representation is insufficient to represent the highly complex multi-class data.

Across datasets, both Geneformer-based models, the cell-sentence approach, and the marker-based baseline are consistently outperformed by the other models, with few task-specific exceptions. CellWhisperer uses Geneformer v1 as an omics embedder and training parameters of the mmContext models were adapted to match the CellWhisperer training. Therefore it remains unclear why there is such a large performance difference between CellWhisperer and the Geneformer v1 model.

The gs10k and the PCA approaches are based on the same selected input genes, the gs10k variant preserves the original feature space while PCA compresses it by dimensionality reduction. This compression by PCA leads to a sharp drop in performance on the tabula sapiens dataset, implying a sustantial loss of information in such a more complex and heterogeneous setting, while performance on the other datasets remain competitive and, in case of the disease classificiation task, even dominant. The scVI-based approach represents a related strategy in which a pretrained nonlinear dimensionality reduction model provides a fixed 50-dimensional latent representation based on a fixed set of 8000 input genes, which serves as the initial representation for mmContext training. In the present benchmark, scVI performs particularly well on broad immune labels and on ranking-based metrics in tabula sapiens, but it does not consistently dominate across datasets.

Overall, Figures S1, S2, S3 and S4 show that the performance ranks of different models are

relatively stable across metrics within each dataset. The mean-rank summary in Figure S5 indicates that the gs10k model provides the best aggregate performance across datasets, supporting the conclusion that a simple gene-selection-based representation can provide a competetive and robust input for multimodal contrastive training.

Table 2: Evaluation results on the *hiha* dataset for the *AIFI_L1* label (broad cell-type annotations).

Model	Bal. Acc.	Baseline Acc. (1/n)	Macro F1	Mean AUC	MRR	Top-1 Acc.	Top-3 Acc.	Top-5 Acc.
gs10k	0.839	0.111	0.539	0.982	0.922	0.863	0.982	0.997
PCA	0.824	0.111	0.663	0.982	**0.962**	**0.928**	0.997	**1.000**
scVI	**0.896**	0.111	**0.702**	0.975	0.950	0.902	**0.998**	1.000
Geneformer	0.763	0.111	0.512	0.989	0.788	0.661	0.902	0.979
Geneformer v1	0.738	0.111	0.516	0.956	0.954	0.923	0.983	0.996
Cell Sentences	0.355	0.111	0.351	0.837	0.816	0.760	0.819	0.841
CellWhisperer	0.744	0.111	0.361	**0.989**	0.670	0.496	0.816	0.983
SCSA	0.528	0.111	0.412	–	–	–	–	–

Table 3: Evaluation results on the *hiha* dataset for the *AIFI_L2* label (fine-grained cell-type annotations).

Model	Bal. Acc.	Baseline Acc. (1/n)	Macro F1	Mean AUC	MRR	Top-1 Acc.	Top-3 Acc.	Top-5 Acc.
gs10k	0.430	0.034	**0.364**	0.920	**0.793**	0.664	**0.912**	**0.936**
PCA	0.365	0.034	0.229	0.907	0.531	0.265	0.762	0.890
scVI	0.418	0.034	0.331	0.880	0.681	0.515	0.856	0.931
Geneformer	0.395	0.034	0.317	0.927	0.791	**0.680**	0.889	0.917
Geneformer v1	0.302	0.034	0.250	0.861	0.676	0.483	0.862	0.922
Cell Sentences	0.166	0.034	0.117	0.745	0.447	0.282	0.493	0.662
CellWhisperer	**0.436**	0.034	0.232	**0.936**	0.567	0.360	0.721	0.854
SCSA	0.100	0.034	0.062	–	–	–	–	–

Table 4: Evaluation results on the *human_disease* dataset for the *Disease* label.

Model	Bal. Acc.	Baseline Acc. (1/n)	Macro F1	Mean AUC	MRR	Top-1 Acc.	Top-3 Acc.	Top-5 Acc.
gs10k	0.081	0.010	0.062	**0.822**	0.210	0.107	0.224	0.304
PCA	**0.105**	0.010	0.063	0.818	**0.266**	**0.150**	**0.297**	**0.387**
scVI	0.038	0.010	0.024	0.645	0.123	0.058	0.106	0.147
Geneformer	0.036	0.010	0.017	0.649	0.120	0.042	0.108	0.162
Geneformer v1	0.029	0.010	0.012	0.642	0.089	0.030	0.068	0.102
Cell Sentences	0.052	0.010	0.020	0.639	0.178	0.075	0.214	0.270
CellWhisperer	0.099	0.010	**0.072**	0.694	0.247	0.140	0.269	0.351

Table 5: Evaluation results on the *human_pancreas* dataset for the *celltype* label.

Model	Bal. Acc.	Baseline Acc. (1/n)	Macro F1	Mean AUC	MRR	Top-1 Acc.	Top-3 Acc.	Top-5 Acc.
gs10k	0.371	0.071	0.198	0.858	0.393	0.252	0.364	0.527
PCA	0.438	0.071	0.295	**0.860**	0.350	0.175	0.379	0.526
scVI	0.433	0.071	0.277	0.790	0.330	0.182	0.326	0.378
Geneformer	0.223	0.071	0.068	0.754	0.263	0.082	0.266	0.381
Geneformer v1	0.291	0.071	0.185	0.772	0.360	0.160	0.439	0.605
Cell Sentences	0.174	0.071	0.067	0.651	0.153	0.025	0.062	0.148
CellWhisperer	**0.461**	0.071	0.273	0.810	**0.532**	**0.348**	**0.637**	**0.758**
SCSA	0.358	0.071	**0.320**	–	–	–	–	–

Table 6: Evaluation results on the *tabula_sapiens* dataset for the *cell_ontology_class* label.

Model	Bal. Acc.	Baseline Acc. (1/n)	Macro F1	Mean AUC	MRR	Top-1 Acc.	Top-3 Acc.	Top-5 Acc.
gs10k	**0.249**	0.006	**0.153**	0.915	0.367	**0.227**	0.415	0.527
PCA	0.013	0.006	0.003	0.567	0.037	0.004	0.015	0.028
scVI	0.170	0.006	0.102	**0.916**	**0.393**	0.225	**0.463**	**0.610**
Geneformer	0.181	0.006	0.101	0.914	0.308	0.173	0.358	0.467
Geneformer v1	0.145	0.006	0.092	0.907	0.334	0.198	0.356	0.482
Cell Sentences	0.066	0.006	0.026	0.818	0.168	0.071	0.185	0.253
CellWhisperer	0.175	0.006	0.101	0.911	0.294	0.153	0.323	0.428
SCSA	0.066	0.006	0.042	–	–	–	–	–

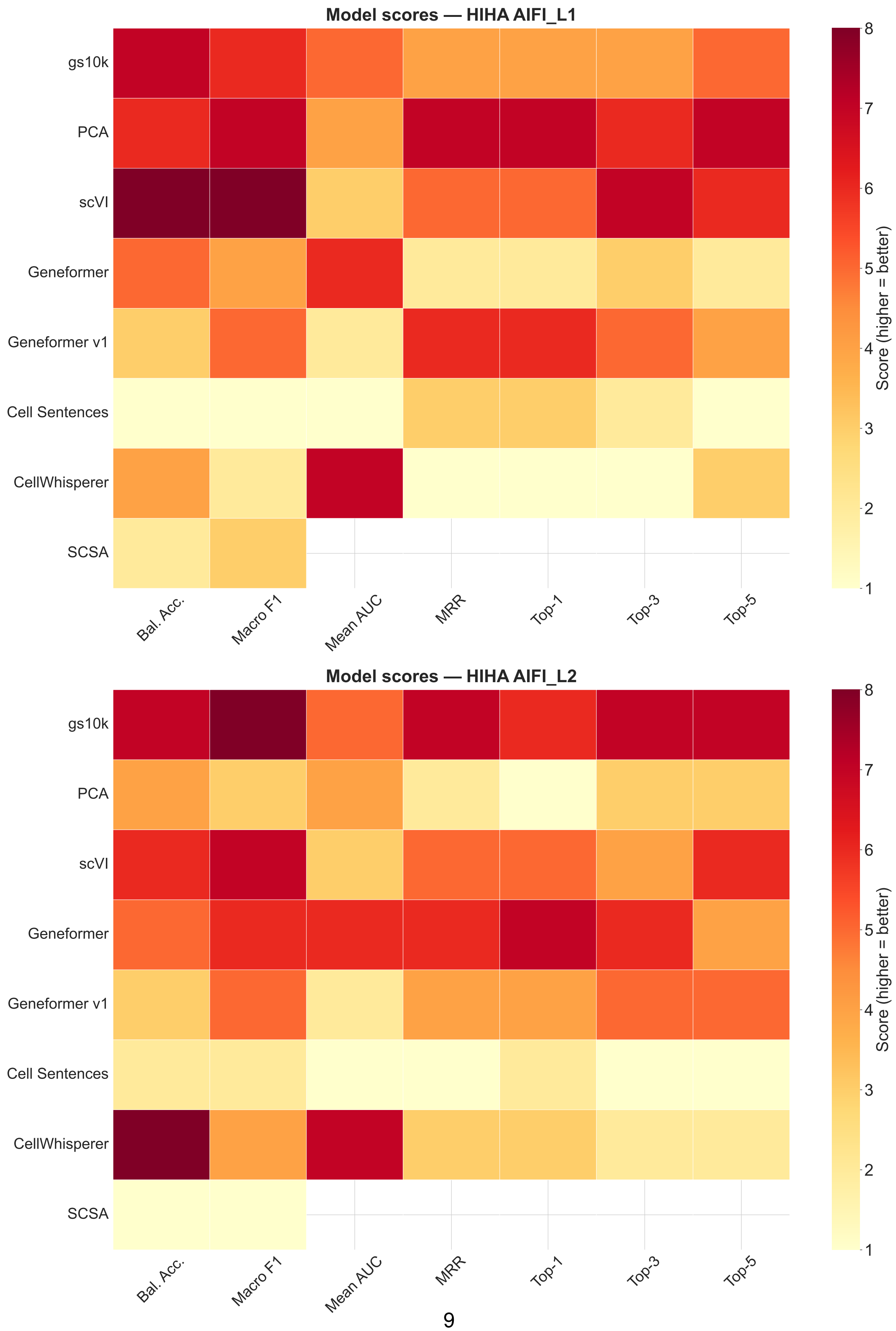

Figure S1: Ranks across metrics on broad (AIFI_L1) and fine-grained (AIFI_L2) annotations of the human immune health atlas.

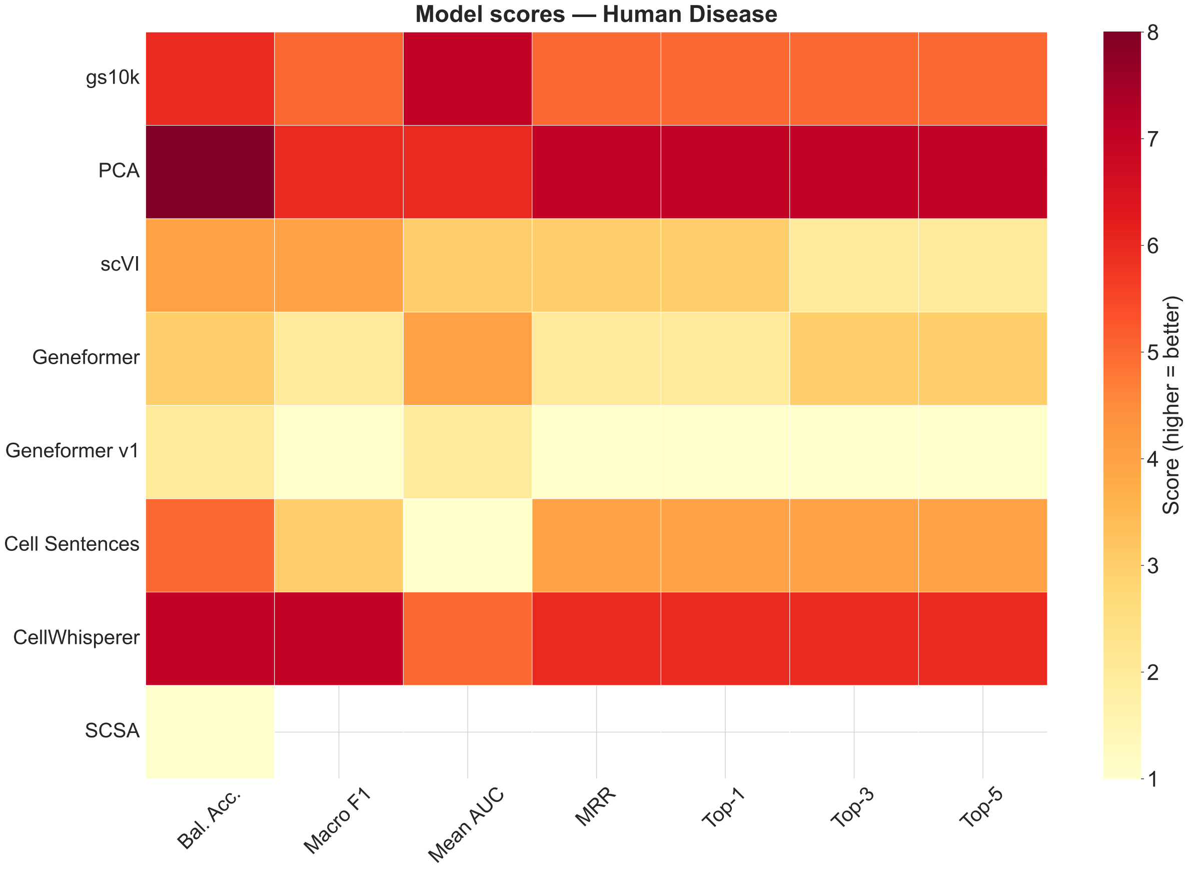

Figure S2: Ranks across metrics on the Human Disease dataset.

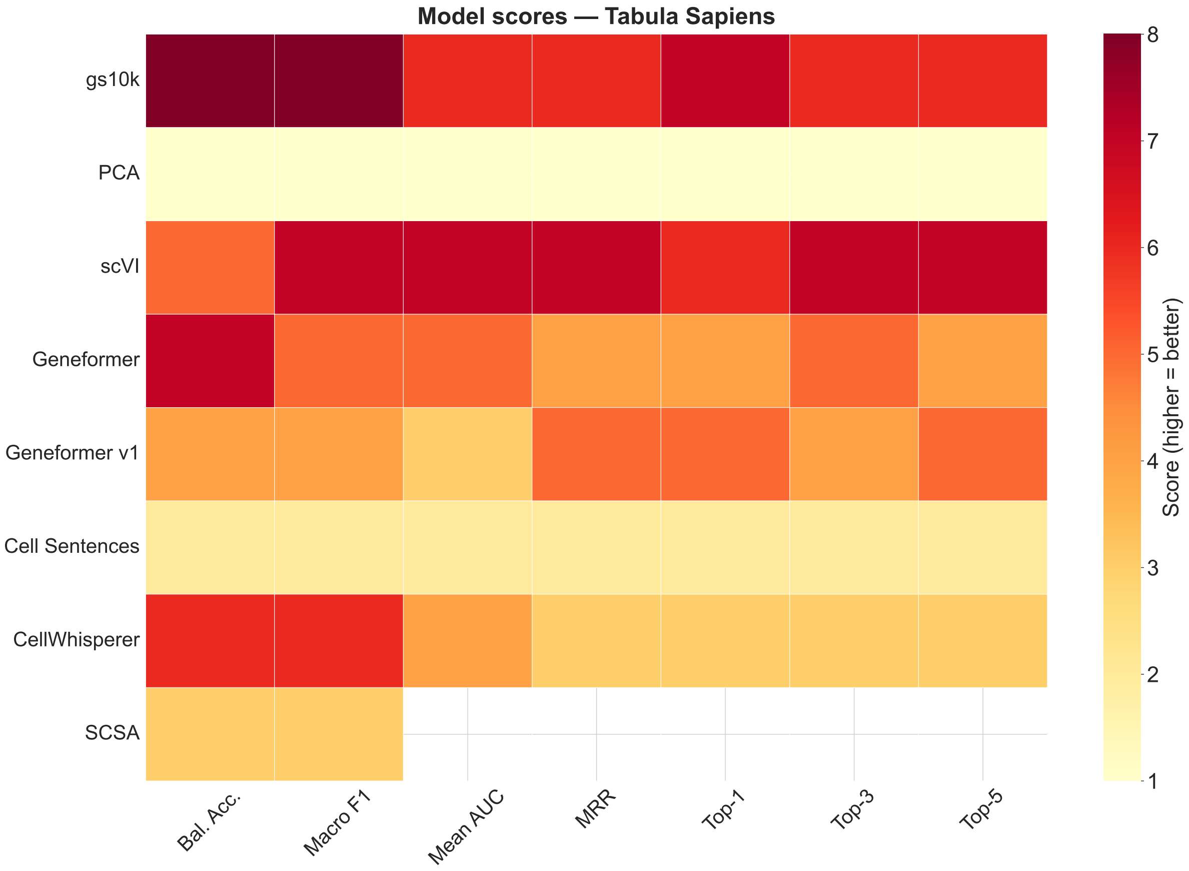

Figure S3: Ranks across metrics on the Tabula Sapiens dataset

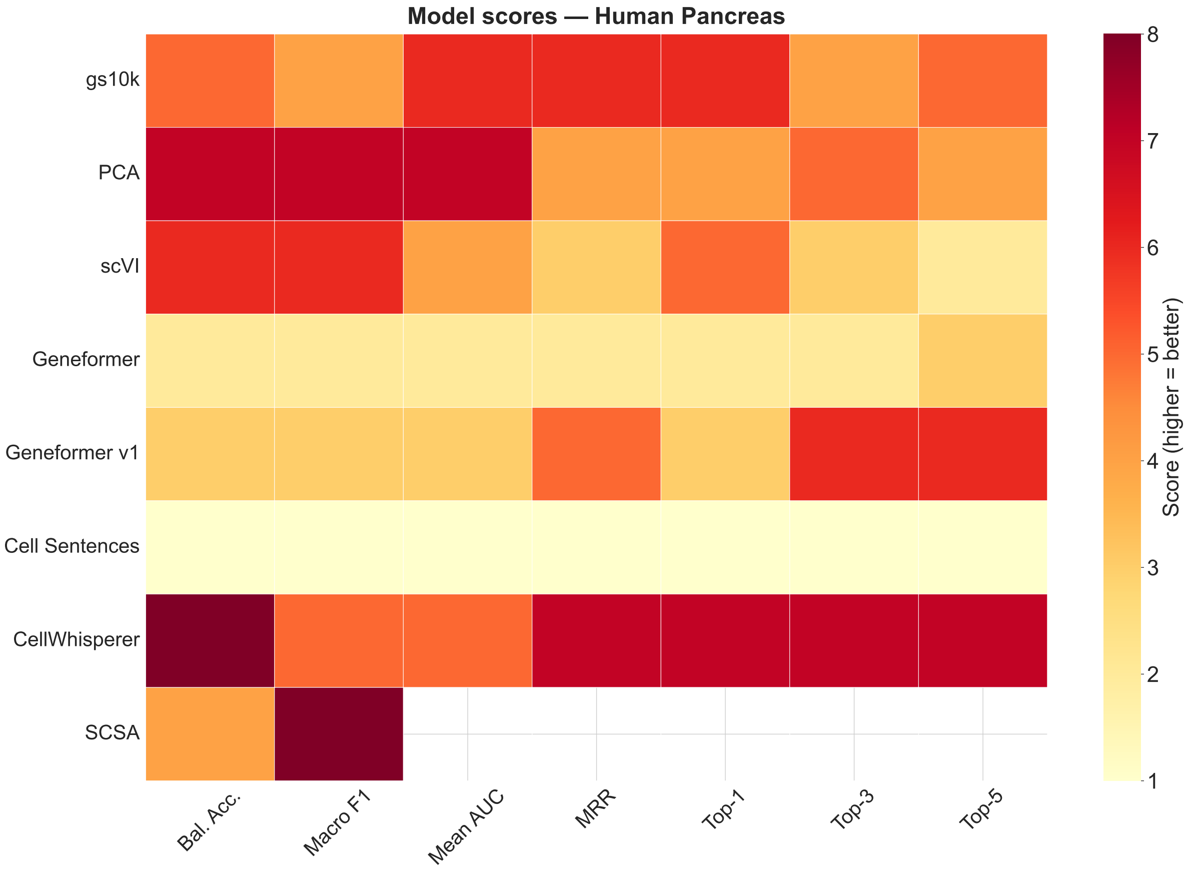

Figure S4: Ranks across metrics on the Human Pancreas dataset.

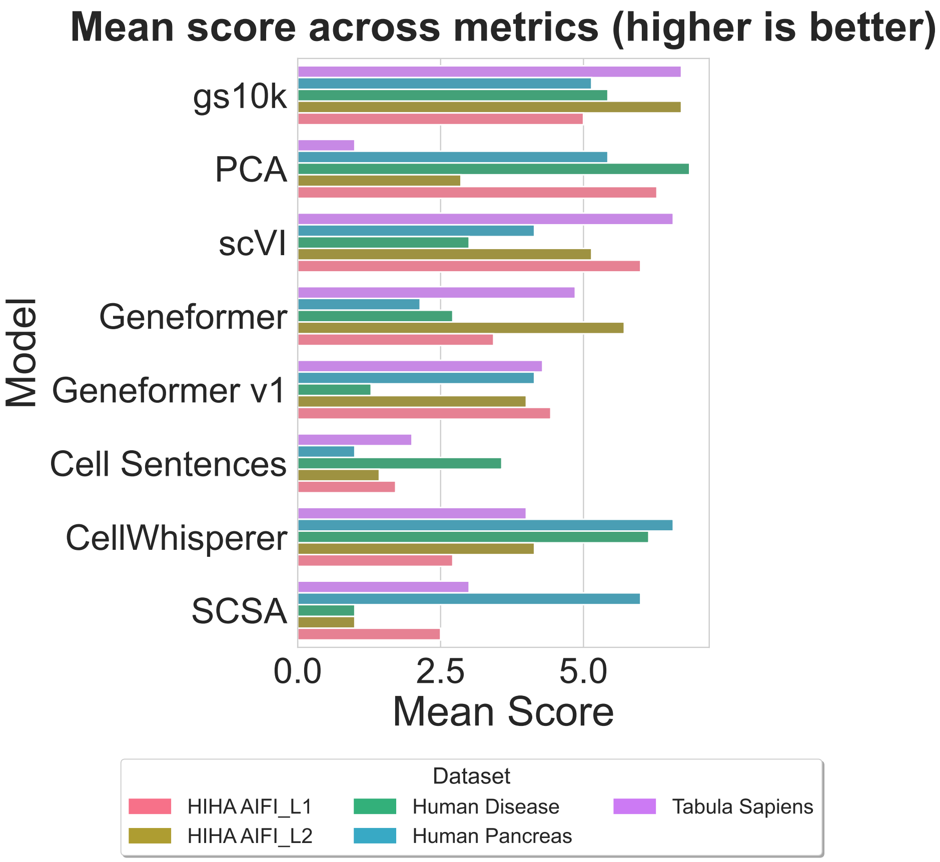

Figure S5: Mean score across metrics for each model and dataset (higher is better). Scores are computed as $n_{\text{models}} + 1 - \text{rank}$, where rank 1 corresponds to the best-performing model.

S7 SCSA Baseline

We included SCSA (Single Cell Subtype Annotation) (Cao et al., 2020) as a non-embedding baseline method for cell-type annotation. Unlike the embedding-based models in our benchmark, which operate by comparing learned cell and label representations in a shared embedding space, SCSA is a marker-gene-based annotation tool that works at the cluster level. It identifies cell types by matching differentially expressed genes against the CellMarker database, a curated repository of experimentally validated cell type markers.

For each dataset, the SCSA pipeline proceeds as follows: (1) Cells are clustered using the Louvain algorithm on a neighborhood graph built from PCA-reduced, highly variable gene expression data. (2) Marker genes are identified per cluster via a t-test for differential expression. (3) SCSA scores each cluster against the CellMarker database, ranking candidate cell types by a Z-score. The top-scoring cell type is assigned to all cells in a cluster. Clusters for which SCSA returns no prediction are labelled "Unknown".

We used Louvain clustering at resolution 1.0 with 2000 highly variable genes selected using the Seurat v3 flavor. SCSA was run with a minimum log fold-change threshold of 1.5 and a maximum p-value threshold of 0.01 for marker gene filtering. The species was set to Human, tissue to All (no tissue-specific filtering), and gene symbols were used as identifiers. The CellMarker v2 database (whole_v2.db) served as the reference.

Because SCSA predictions use free-text cell type names from CellMarker (e.g., "Alpha cell (α cell)") that differ from the ground-truth label vocabularies of the benchmark datasets (e.g., "pancreatic A cell"), a label harmonization step is required before metrics can be computed. We used calmate, a tool that maps free-text cell type labels to standardised Cell Ontology terms using semantic similarity.Menger, 2026 Calmate was applied independently to both the SCSA-predicted labels and the ground-truth labels, producing a shared ontology-backed vocabulary. Each mapping was manually reviewed and corrected where necessary using calmate's interactive review mode. Metrics were then computed on the harmonised labels.

Since SCSA produces hard cluster-level predictions rather than continuous similarity scores, only classification-based metrics (accuracy, balanced accuracy, macro F1) are reported. Embedding-based metrics such as mean AUC, top-k accuracy, and MRR are not applicable and are omitted.

S8 Usage of LLMs

Large Language models were utilized to write and improve code and documentation as well as to improve the manuscript. howpublished = https://github.com/mengerj/calmate,

S9 References

1. Barrett T, Suzek TO, Troup DB, Wilhite SE, Ngau WC, Ledoux P, Rudnev D, Lash AE, Fujibuchi W, and Edgar R. NCBI GEO: mining millions of expression profiles—database and tools. Nucleic acids research 2005;33:D562–D566.
2. Biewald L. Experiment Tracking with Weights and Biases. Software available from wandb.com. 2020. URL: https://www.wandb.com/.
3. Cao Y, Wang X, and Peng G. SCSA: a cell type annotation tool for single-cell RNA-seq data. Frontiers in genetics 2020;11:490.

4. CZI Cell Science Program, Abdulla S, Aevermann B, Assis P, Badajoz S, Bell SM, Bezzi E, Cakir B, Chaffer J, Chambers S, et al. CZ CELLxGENE Discover: a single-cell data platform for scalable exploration, analysis and modeling of aggregated data. Nucleic acids research 2025;53:D886–D900.
5. Ergen C, Pour Amiri VV, Kim M, Kronfeld O, Streets A, Gayoso A, and Yosef N. Scvi-hub: an actionable repository for model-driven single-cell analysis. Nature Methods 2025:1–10.
6. Henderson M, Al-Rfou R, Strope B, Sung YH, Lukács L, Guo R, Kumar S, Miklos B, and Kurzweil R. Efficient natural language response suggestion for smart reply. arXiv preprint arXiv:1705.00652 2017.
7. Levine D, Rizvi SA, Lévy S, Pallikkavaliyaveetil N, Zhang D, Chen X, Ghadermarzi S, Wu R, Zheng Z, Vrkic I, et al. Cell2Sentence: teaching large language models the language of biology. BioRxiv 2024:2023–9.
8. Menger J. calmate: Cell Annotation Label Mapping with Assisted Term Editing. https://github.com/mengerj/calmate. Accessed: 2026-03-18. 2026.
9. NeuML. PubMedBERT Embeddings. https://huggingface.co/NeuML/pubmedbert-base-embeddings/tree/main. Accessed: 2025-11-03. 2023.
10. Reimers N and Gurevych I. Sentence-BERT: Sentence Embeddings using Siamese BERT-Networks. In: *Proceedings of the 2019 Conference on Empirical Methods in Natural Language Processing*. Association for Computational Linguistics, 2019. URL: http://arxiv.org/abs/1908.10084.
11. Rizvi SA, Levine D, Patel A, Zhang S, Wang E, He S, Zhang D, Tang C, Lyu Z, Darji R, et al. Scaling Large Language Models for Next-Generation Single-Cell Analysis. bioRxiv 2025:2025–4.
12. Schaefer F, Cannoodt R, Sikkema L, Eraslan G, and Theis FJ. Multi-modal foundation models for scalable single-cell analysis. bioRxiv 2024.
13. Theodoris CV, Xiao L, Chopra A, Chaffin MD, Al Sayed ZR, Hill MC, Mantineo H, Brydon EM, Zeng Z, Liu XS, et al. Transfer learning enables predictions in network biology. Nature 2023;618:616–24.
14. Virshup I, Rybakov S, Theis FJ, Angerer P, and Wolf FA. anndata: Access and store annotated data matrices. Journal of Open Source Software 2024;9:4371.
